# The outcomes of consecutive pregnancies in Australian women with epilepsy

**DOI:** 10.1002/epd2.70265

**Published:** 2026-05-15

**Authors:** Frank Vajda, Terence J. O'Brien, Janet Graham, Alison Hitchcock, Piero Perucca, Cecilie Lander, Simon Vajda, Mervyn Eadie

**Affiliations:** ^1^ Departments of Medicine and Neurosciences The Royal Melbourne Hospital, the University of Melbourne Parkville Victoria Australia; ^2^ Department of Neuroscience The School of Translational Medicine, Monash University Melbourne Victoria Australia; ^3^ Department of Neurology Alfred Health Melbourne Victoria Australia; ^4^ Department of Medicine Austin Health, the University of Melbourne Heidelberg Victoria Australia; ^5^ Bladin‐Berkovic Comprehensive Epilepsy Program, Austin Health Heidelberg Victoria Australia; ^6^ Royal Brisbane and Women's Hospital and School of Medicine and Biomedical Science, University of Queensland Brisbane Queensland Australia; ^7^ Applied Artificial Intelligence Initiative, Deakin University Burwood Victoria Australia

**Keywords:** antiseizure medication, consecutive pregnancies, fetal malformation, focal epilepsy, generalized epilepsy, seizure freedom

## Abstract

**Objective:**

To investigate the extent to which seizure control and the occurrence of fetal malformation in an initial pregnancy can serve to anticipate the outcome in the next pregnancy.

**Methods:**

We analyzed the records of the Raoul Wallenberg Australian Register of Antiepileptic Drugs in Pregnancy for seizure control and fetal malformation data for women with epilepsy taking antiseizure medication in consecutive pregnancies.

**Results:**

Seizure freedom rates throughout pregnancy were higher in the subsequent pregnancy than in the initial one (60.8% vs. 52.9%; RR 1.15, 95% CI 1.03, 1.28), particularly when the nature of antiseizure therapy was unchanged between pregnancies. Fetal malformation occurrence rates (5.9% and 6.2%, respectively) were similar, but tended to be higher in women with generalized rather than focal epilepsies (7.9% vs. 4.28%; RR 1.84, 95% CI 0.89, 3.81). If a small cohort with fetal malformations in both the initial and subsequent pregnancies (7 women, 8 pairs of pregnancies) was excluded, the RR value was smaller (1.24). All but one woman in this cohort had generalized epilepsies. The birth of a malformed baby tended to occur more frequently in the next pregnancy for women with generalized epilepsy and a malformed baby in the previous pregnancy than in similar women with focal epilepsies (50% vs. 7.7%; RR 6.5, 95% CI 0.92, 45.1).

**Significance:**

Compared with women with focal epilepsies, antiseizure medication‐treated women with generalized epilepsy who give birth to a malformed baby appear to have a substantially greater risk of another malformed baby in their next pregnancy. Overall, seizure freedom rates were higher in subsequent pregnancies than in initial ones.

**Key points**

- ASM‐treated consecutive pregnancies in WWE were investigated.
- Seizure freedom and malformations were evaluated.
- Higher seizure freedom rates in the second of consecutive pregnancies.
- Higher risk of malformations with generalized epilepsy, possibly genetic.
- Consecutive pregnancy malformation rates are reasonably similar if ASMs are unchanged.

## 1 INTRODUCTION

Over its 25‐year existence, the Australian‐based Raoul Wallenberg Register of Antiseizure Medication in Pregnancy (APR) has been primarily concerned with obtaining locally applicable data regarding the extent to which intra‐uterine exposure to antiseizure medications (ASMs) in women with epilepsy (WWE) was associated with the occurrence of fetal malformation (FM). Additional potentially relevant information was also obtained, especially data concerning epileptic seizure control in pregnancy. More recently, the matter of intrauterine exposure to ASM and subsequent adverse neurocognitive development during childhood has been pursued.

An appreciable number of WWE have had more than one pregnancy included in the APR database. We have previously attempted^1^, ^2^ to analyze the FM data from such repeated pregnancies in the same woman using the data available at earlier stages of the APR's existence, and have dealt separately with the potentially related matter of seizure control in them.^3^ Utilizing a more recent and expanded APR data set, the following material attempts to investigate both FM and seizure control, but only in WWE who have consecutive pregnancies rather than simply any two or more pregnancies included in the current APR data set. The analysis takes a particular interest in whether events in the initial pregnancy may predict what occurs in the succeeding one.

## 2 MATERIALS AND METHODS

### 2.1 The APR

The present paper is based on pregnancies drawn from the APR's records as of April 2025. More complete details of the APR's activities and its recruitment policies and practices have been published previously.^4^, ^5^ The Australian WWE involved in the APR had either been treated with ASMs before and throughout pregnancy or, considerably less often, had not taken these drugs in at least the earlier months of pregnancy, though some in this subgroup may have taken ASMs before pregnancy. The inclusion of WWE was always on a voluntary basis, after they knew of the APR's existence and purpose. All women enrolled have provided written informed consent to participation. The APR has been estimated to contain data concerning about 8.7% of the relevant pregnancies of WWE that occur in Australia.^4^, ^5^

After the initial contact, however achieved, all further contact between the pregnant woman and the Melbourne‐based APR office has been by telephone. Data, principally that concerning the progress of the pregnancy and the control of the woman's seizure disorder, are collected from the women at (i) enrolment, (ii) approximately 28 weeks of pregnancy, (iii) in the weeks immediately following the end of the first post‐partum month, and, if possible, (iv) a year after the end of the pregnancy. The accuracy of the information provided by the WWE depends on the reliability of their recall, though it was checked with the records of their treating medical practitioners whenever feasible. Clinical management always remained in the hands of those practitioners. During its existence, the APR has been under the ethics oversight of several Melbourne‐based institutional Human Ethics Research Committees, currently that of Melbourne Health.

### 2.2 The pregnancies studied

Pregnancies in WWE with unknown FM or seizure control outcomes were excluded from further consideration. By April 2025, the APR contained records of 2749 completed pregnancies in Australian WWE. In 1057 of these pregnancies, 472 of the WWE had experienced at least two consecutive pregnancies (in 91, three; in 19, four; and in 3, five). APR data are recorded anonymously in relation to individual pregnancies, with the pregnancies in the same woman linkable through individual separate identification numbers. To be accepted as having carried at least two APR pregnancies, a WWE was further required to have identical dates of birth in all her APR records.

### 2.3 Criteria evaluated

Epileptic seizure control was assessed in terms of (i) complete seizure freedom in the pre‐pregnancy year and (ii) complete seizure freedom throughout pregnancy. Such seizure freedom provides the main determinant of limitation to way of life in WWE capable of pregnancy, particularly in relation to driving motor vehicles.

FMs were categorized according to the Riley et al. criteria.^6^ These rates of FM occurrence were those that applied at the end of the post‐natal year. In the few instances where the woman concerned could not be contacted at that stage, the rates used were those that applied at her post‐natal interview.

### 2.4 Data analysis

Data relevant to the present study were extracted from the APR database into an Excel spreadsheet, and then analyzed using conventional simple statistical techniques, principally confidence interval analysis and simple linear regression utilizing Stats Direct software. Throughout, a *p* < 0.05 level of confidence was considered statistically significant.

## 3 RESULTS

### 3.1 The women and their pregnancies

Table 1 provides data characterizing the women studied and their pregnancies recorded in the APR database.

**TABLE 1:** Characteristics of the women studied and their pregnancies.

Pregnancy	1	2	3	4	5
Number of WWE	472	472	91	19	3
Age ± S.D. (years)	29.96 ± 3.84	32.38 ± 4.22	33.43 ± 3.91	36.32 ± 3.71	38
Primiparous	332 (70.6%)	‐	‐	‐	‐
Epilepsy					
Generalized	45.3%	45.3%	48.4%	31.6%	0%
Focal	50.6%	50.6%	51.6%	68.4%	100%
Onset age (years)	17.04 ± 7.40	17.04 ± 7.40	15.65 ± 6.87	15.84 ± 6.43	25.33
Duration (years)	12.86 ± 7.64	15.40 ± 7.99	18.84 ± 6.90	20.47 ± 7.34	12.67
Enrolment source					
Neurologist	64.5%	2.3%	2.2%	0%	0%
Other medical	10.8%	0.8%	1.1%	5.3%	0%
Self (re)‐referral	0.8%	96.6%	96.7%	94.7%	100%
Pregnancy outcome					
Live birth	93.2%	95.3%	94.5%	100%	100%
Twins	0.6%	3.4%	2.2%	0%	0%
ASM Use					
None	7.2%	7.6%	5.5%	0%	0%
Monotherapy	68.4%	71.1%	74.4%	78.9%	100%
CBZ	27.2%	27.4%	21.9%	40.0%	66.7%
LTG	30.0%	31.0%	32.8%	20.0%	0%
LEV	16.4%	19.4%	12.5%	13.3%	33.3%
TPM	3.7%	3.9%	4.7%	6.7%	0%
VPA	16.7%	14.2%	15.6%	6.7%	0%
Mono‐ or polytherapy					
CBZ	29.5%	28.4%	25.6%	52.6%	66.7%
LTG	35.6%	35.1%	38.4%	21.1%	0%
LEV	22.1%	26.4%	15.1%	10.5%	33.3%
TPM	7.8%	7.6%	4.7%	5.3%	0%
VPA	20.1%	17.9%	19.8%	5.3%	0%

Abbreviations: CBZ, carbamazepine; LEV, levetiracetam; LTG, lamotrigine; TPM, topiramate; VPA, valproate.

The rather striking difference (almost 30‐fold) between the first and subsequent pregnancies in relation to the extent of referral to the APR by neurologists and that by the WWE themselves seems surprising at first sight. However, some 96% of a random sample (*N* = 50) of the self‐referred second pregnancy women nominated individual Australian neurologists as the current source of provision of information concerning their seizure disorders during their self‐referred pregnancies.

### 3.2 Seizure control

Seizure freedom occurrence rates (Table 2) seem largely unchanged between the pre‐pregnancy year and throughout pregnancy and labor, irrespective of the number of pregnancies in the individual WWE. The increasing seizure freedom rate in the post‐partum weeks, with an increasing number of pregnancies in individual women, seems to have occurred at a time of expected increasing experience in managing personal life activities while accommodating a new baby into a household. The decreased frequency of pre‐pregnancy changes made in the ASM(s) taken with increased pregnancy number may relate to decreasing options for better seizure control or more effective avoidance of foetal FM.

**TABLE 2:** Seizure freedom rates at different times relative to pregnancy regressed on the number of APR pregnancies in the individual WWE.

Pregnancy	1	2	3	4	5	*p* =
Number of WWE	472	472	91	19	3	
Pre‐pregnancy ASM change	22.7%	11.9%	7.7%	5.0%	0%	.008
Seizure free						
Pre‐pregnancy year	54.0%	68.65%	59.3%	63.2%	66.7%	.349
Pregnancy	52.5%	62.1%	60.4%	57.9%	33.3%	.304
Labor	98.1%	89.2%	98.9%	100%	100%	.575
Postpartum weeks	81.6%	85.6%	84.6%	94.7%	100%	.018

*Note*: The *p* values relate to the linear regression slope.

### 3.3 Fetal malformation

The hazard of the occurrence of a malformed fetus in the pregnancies overall appeared greater, though not statistically significantly so, if there had been intrauterine ASM exposure than if there had been none, at least in the earlier half of pregnancy (63/982 vs. 2/75, i.e., 6.42% vs. 2.67%; RR 2.41, 95% CI 0.60, 9.64).

The overall malformed fetus occurrence rate did not alter statistically significantly despite progressively falling rates of freedom from seizures in the pre‐pregnancy year and throughout pregnancy as the number of pregnancies in the individual WWE increased (Table 3). There was also no statistically significant change in the apparently mildly increasing occurrence rates for giving birth to a structurally intact fetus. The latter was a situation where seizure freedom rates remained relatively static as pregnancy number in the individual WWE increased.

**TABLE 3:** Fetal malformation occurrence and non‐occurrence rates, and seizure full control correlations before and through pregnancy, and the pattern of ASM use regressed on the number of APR pregnancies in the individual WWE.

Pregnancy	Preg 1	Preg 2	Preg 3	Preg 4	Preg 5	*p* =
Number of pregnancies	472	472	91	19	3	
*Malformed fetus*	30 (6.4%)	29 (6.1%)	6 (6.6%)	1 (5.5%)	0 (0%)	.125
Associated seizure control						
Seizure freedom in						
Pre‐pregnancy year	60.0%	41.4%	16.7%	0%	0%	.008
Through pregnancy	56.7%	34.5%	33.3%	0%	0%	.013
*Non‐malformed fetus*	442 (93.6%)	443 (93.9%)	85 (93.4%)	18 (94.7%)	3 (100%)	.095
Associated seizure control						
Seizure freedom in						
Pre‐pregnancy year	53.6%	70.4%	58.2%	66.7%	66.7%	.378
Through pregnancy	52.3%	63.9%	58.2%	61.1%	33.3%	.360
ASM use in pregnancies involving malformations						
No use	3.3%	3.4%	0%	0%	0%	.061
Use in	96.7%	96.6%	100%	100%	100%	.061
Monotherapy	75.9%	65.5%	50.0%	77.8%	100%	.367
Mono or polytherapy	24.1%	32,1%	50.0%	22.2%	0%	.382
CBZ*	24.1%	25.0%	50.0%	52.6%	66.7%	.010
LTG*	27.6%	25.0%	16.7%1	21.1%	0%	.064
LEV*	10.3%	28.6%	16.7%	10.5%	33.3%	.797
TPM*	10.3%	10.7%	16.7%	5.3%	0%	.231
VPA*	41.4%	28.6%	16.7%	5.3%%	0%	.001

*Note*: *p* values relate to linear regression slopes.

* As a % of total ASMs use.

The decreasing likelihood of FM with growing numbers of pregnancies in the individual WWE (Table 3) seemed to be associated with increasing rates of intrauterine exposure to CBZ and decreasing rates of exposure to VPA.

### 3.4 Consecutive pregnancies

In this and the following Results subsections, the few 4th and 5th consecutive pregnancies, the initial consecutive pregnancies not exposed to ASM therapy, and those resulting from assisted fertilization^7^ were excluded from consideration. The initial pregnancies that are dealt with further in the paper comprised the remaining firstst pregnancies and those second pregnancies which were followed by a third pregnancy (total = 488). The subsequent pregnancy set (total = 498) comprised all the remaining pregnancies, mainly the second ones.

#### 3.4.1 Seizure freedom through pregnancy

Of the 488 members of the initial pregnancy set, 258 remained seizure‐free throughout pregnancy (52.9%). The corresponding seizure freedom rate in the next pregnancy set was higher (303/498, i.e., 60.8%; RR = 1.15; 95% CI 1.03, 1.28). A higher seizure freedom rate in the next pregnancies also applied for both generalized and focal epilepsy sufferers (Table 4). There had been changes in the ASMs employed between consecutive pregnancies in 72 instances, including 37 where valproate was the drug involved, its dose being reduced in 16 and its intake ceased in another 21. Where the ASMs were unchanged between consecutive pregnancies, the seizure freedom rate throughout pregnancy was statistically significantly higher than when there had been ASM changes (Table 4). This outcome also tended to apply for both generalized and focal epilepsies, and to a statistically significant extent in relation to the latter group.

**TABLE 4:** Comparisons between the initial and the subsequent pregnancy for seizure control and fetal malformation occurrence in consecutive pregnancies in the same WWE, and the effects of ASM change between pregnancies. The data apply to ASM‐treated pregnancies where assisted fertilization was not involved. RR = risk ratio.

	Initial Preg.	Subsequent Preg.		RR	95% CI
Seizure‐free pregnancy occurrence					
	258/488 (52.9%)	303/498 (60.8%)		1.15	1.03, 1.28
If ASM changed		19/44 (43.2%)	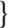	0.69	0.49, 0.98
If ASM unchanged		284/454 (62.6%)			
Generalized epilepsies	134/220 (60.9%)	149/229 (65.1%)		1.07	0.93, 1.23
If ASM changed		12/26 (46.2%)	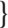	0.68	0.47, 1.05
If ASM unchanged		137/203 (67.5%)			
Focal epilepsies	115/248 (46.4%)	148/257 (57.6%)		1.24	1.05, 1.47
If ASM changed		7/18 (38.9%)	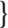	0.66	0.37, 1.19
If ASM unchanged		140/238 (58.8%)			
Malformation occurrence					
Overall	30/488 (6.2%)	29/498 (5.9%)		0.95	0.58, 1.56
If ASM changed		1/44 (2.3%)	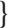	0.37	0.05, 2.64
If ASM unchanged		28/454 (6.2)			
Occurred in initial pregnancy		8/29 (27.6%)	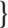	6.16	2.99, 12.69
Absent in the initial pregnancy		21/469 (4.7%)			
With ASMs unchanged between pregnancies					
Occurred in initial pregnancy		8/29 (27.6%)	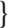	5.86	2.83, 12.15
Absent in the initial pregnancy		20/425 (4.7%)			
Generalized epilepsies		18/229 (7.9%)	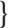	1.84	0.89, 3.81
Focal epilepsies		11/257 (4.1%)			

*Note*: The numbers of initial and subsequent pregnancies are greater than the number of women carrying first pregnancies because some second pregnancies are initial pregnancies in relation to subsequent third pregnancies. The type of epilepsy could not be categorized in occasional pregnancies.

#### 3.4.2 Malformed fetus occurrence

The malformed fetus occurrence rates in the ASM‐exposed pregnancies (Table 4) were 30/488 for the initial pregnancies and 29/498 for subsequent ones (6.2% vs. 5.9%; RR = 0.95, 95% CI 0.58, 1.56). Where the nature of the ASMs used had changed between pregnancies, the FM occurrence rate tended to be lower, though not statistically significantly so, in the next pregnancies compared with when the ASMs used were unchanged (2.3% in 44 vs. 6.2% in 454; RR = 0.37; 95% CI 0.05, 2.64).

Malformed fetuses occurred in the pregnancy in 8 of the 30 initial pregnancies in which FM had occurred. All the 8 women involved were primiparous in their initial pregnancies. In the next pregnancies, their 8 FMs comprised 27.6% of the total of 29 FMs that had occurred. The remaining 21 WWE with FMs comprised 4.7% of the remaining non‐malformed 469 pregnancies (RR 6.16; 95% CI 2.99, 12.69). It was not possible to assess whether a change in ASM between pregnancies might have altered this increased hazard of a next pregnancy carrying a malformed fetus if the initial pregnancy outcome was malformed. There were no changes in ASM between pregnancies in any of the 8 sets of consecutive pregnancies being considered. When the full next pregnancy, the FM rate of 5.8% was used as the comparator in relation to these 8 pregnancies (Table 4), the RR value remained raised (4.74; 95% CI 2.38, 9.42).

The 8 instances of FMs in the next pregnancies included one woman who had a further FM in her third pregnancy. All three fetuses had been exposed in utero to LEV plus TPM. All the malformations were instances of hypospadias, which were also present in her husband, raising the possibility that genetic factors may have been involved. Relevant to this possibility, in both of the affected consecutive offspring of another woman who took VPA in ASM monotherapy, an extra thumb was present. In the remaining women, different malformations had occurred in each of the paired consecutive pregnancies, VPA monotherapy being involved in three women, VPA plus LTG in another, and CBZ monotherapy in 2.

There were also two women in whom similarly malformed fetuses had occurred in their initial and third pregnancies, the malformations being hydronephrosis in one and skull synostosis in the other, the latter malformation having also occurred in other family members. Exposure to CBZ monotherapy was involved in both instances.

Having had a malformed fetus in an initial pregnancy did not appear to necessarily deter WWE from undertaking further pregnancies. There were further pregnancies recorded in the APR in 30% of the WWE, with initial pregnancies involving malformations, but in 17.2% of those without malformations. However, there is no certainty that all subsequent pregnancies, particularly in the latter group, were recorded in the APR.

#### 3.4.3 Outcome prediction possibilities

Seizure freedom occurred a little more often in the next than in the initial consecutive ASM‐treated pregnancies overall, a finding that tended to apply for both generalized and focal epilepsies. Higher seizure freedom rates were present when no ASM changes between pregnancies had occurred (Table 4). Some 86.5% of seizure‐free initial pregnancies were followed by a seizure‐free next pregnancy.

Table 5 compares certain characteristics of the 8 pregnancies with FMs in both initial and next pregnancies with those in which the next pregnancy was malformation free. The obvious, and also statistically significant, differences were in the higher incidence of successive malformations in the ASM‐treated WWE with generalized epilepsies and with family histories of FM. Women with ASM‐exposed generalized epilepsies and a malformed fetus seemed to have a greater risk of a FM in their next pregnancy than women with focal epilepsies (50% vs. 6.7%; RR 7.5, 95% CI 1.05, 53.5).

**TABLE 5:** Comparison of fetal malformation‐associated initial pregnancies between WWE whose fetus was, or was not, malformed in their next pregnancy.

	Malformation in the next pregnancy	No malformation in the next pregnancy	*p*
Number	7	22	
Age in pregnancy – years	29.29 ± 4.39	31.27 ± 3.44	> .10
Epilepsy onset age – years	13.29 ± 6.55	19.05 ± 8.14	> .10
Focal epilepsy	1 (14.3%)	14 (63.6%)	.029
Generalized epilepsy	6 (85.7%)	7 (31.8%)	.026
Family history of epilepsy	4 (57.1%)	6 (27.3%)	.193
1 year with no pre‐pregnant seizures	4 (57.1%)	8 (36.4%)	.403
Seizure‐free pregnancy	3 (42.9%)	9 (40.9%)	1
Family history of fetal malformation	3 (42.9%)	0	.010
ASM change before pregnancy	0	4 (18.2%)	.546
Exposed to:			
Valproate	4 (57.1%)	7 (31.8%)	.375
Carbamazepine	2 (28.6%)	6 (27.3%)	1
Topiramate	1 (14.3%)	2 (9.1%)	1

*Note*: The second pair of consecutive pregnancies in the same woman is omitted from the data. *p* values obtained from Fisher's “exact” test, except for the age data.

## 4 DISCUSSION

Several of the above findings in consecutive pregnancies in women with ASM‐treated epilepsies seem worth discussing further.

There was a progressively decreasing proportion of pregnancies associated with prior changes in the ASMs used as women underwent increasing numbers of pregnancies. This decrease was not associated with any significant change in the subsequent seizure‐free pregnancy occurrence rate. This finding may simply reflect the possibility that any change in the ASM nature made before the first pregnancy under consideration may have already achieved the maximum possible benefit for seizure control in the women concerned.

Even under the management of neurologists (in the great majority of instances), there was relatively little increase in seizure‐freedom rates between initial and subsequent consecutive pregnancies, though no overall decrease. This finding may be a consequence of priority being given to the avoidance of FM and thus limiting the remaining seizure treatment options. It needs to be appreciated that over the quarter of a century during which data used in the present study were collected, additional ASMs became available in Australia, and preferences for the choice of drug employed in pregnancy and in different types of seizure disorder progressively altered. As a result, some of the ASM patterns of use described in this paper may seem inappropriate to contemporary standards.

It seemed that the number of pregnancies that had occurred in the individual WWE was not a major factor in determining the FM occurrence risk. The FM occurrence rates also remained virtually static despite statistically significant overall decreasing rates of intrauterine exposure to valproate and increasing rates of carbamazepine exposure with increasing numbers of pregnancies in the individual WWE. This relatively static rate was also associated with statistically significant falling rates of seizure freedom before and throughout pregnancy. However, when the fetus was morphologically intact, there were no significant changes in seizure freedom rates. In relation to valproate, these findings are consistent with avoidance of FM receiving precedence over full seizure control: in relation to carbamazepine, this ASM possessed a lesser degree of teratogenic capacity than VPA but a reasonable capacity to achieve seizure control. Overall, changing the nature of ASM between pregnancies was found to be associated with a statistically significant reduction in the likelihood of a seizure‐free pregnancy occurring and with a non‐statistically significant reduction in the risk of FM.

In 2013 we,^1^ and also Campbell et al.,^8^ reported an increased risk of FM in a subsequent pregnancy in WWE if there had been a malformation in a previous one. In the present study, when a malformed fetus occurred in an initial pregnancy, there was an approximately six‐fold increased risk of a malformed fetus resulting from the immediately following one, irrespective of whether or not ASMs had been changed between pregnancies (Table 4). Whether this heightened risk would still have applied if ASM therapy had been changed between consecutive pregnancies, which both produced malformed fetuses, could not be determined from the data, since no relevant instances existed in the material studied. It is noteworthy that in nearly half of the pregnancies in which malformed offspring had occurred in consecutive pregnancies, the same malformations had been involved in both pregnancies. This suggests that, as others have also suggested,^9^, ^10^, ^11^, ^12^, ^13^ a genetic factor, either acting alone or in conjunction with the effects of an ASM, may have contributed to the teratogenesis in these pregnancies.

It would obviously be desirable to have available more extensive data regarding the matters investigated in this paper, and also information concerning the degree of hazard of later childhood neurodevelopmental disadvantages that may occur. Unfortunately, it may take a substantial amount of time for such material to become available in the APR dataset. Over this time, aspects of medical practice may continue to change as knowledge grows. Nevertheless, it seems likely that the increased hazard of giving birth to a malformed baby in the following pregnancy will remain if a malformed baby has occurred in the preceding pregnancy. If the mother has a genetic generalized epilepsy, the risk will be even higher. And despite the lack of evidence that the present study can provide, it seems clinically prudent to suggest that changes in ASM therapy should receive serious consideration if a malformed baby has already resulted from an ASM‐exposed pregnancy.

Limitations: There are some difficulties and uncertainties in interpreting the findings of the present study, based as it is on consecutive pregnancies in the same woman. We have no proof that the same biological father has always been involved in the conception of the individual consecutive offspring. Subject numbers are relatively small despite having been collected over a substantial period of time. The WWE studied may not necessarily be representative of the population of Australian WWE, though the APR FM occurrence rate of 2.67% in women with untreated epilepsy was reasonably similar to the national malformation rate of around 3% in 2017 data.^14^ During the period of APR data collection, aspects of medical and therapeutic practice have changed, for instance, in relation to actions prompted by recognition of the malformation hazard associated with intrauterine VPA exposure. While the effects of exposures to different ASMs in consecutive pregnancies were taken into account in the present study, the possible consequences of a changed dosage of the same ASM were not. There simply were too many possibilities relative to the number of relevant pregnancies in the APR database. There is the further issue of how adequately the women involved in the present study are representative of Australian WWE who become pregnant. Recruitment into the study was on a voluntary basis, so that the women involved have, in effect, selected whether they will participate in it. The Australian register was instigated and is managed by neurologists and the great majority of the women involved in it seem able to nominate neurologists among their medical contacts. Hence their epilepsies may have tended to be managed particularly skillfully. The WWE involved have tended to often begin their reproductive careers relatively late and frequently already had well‐established seizure disorders for which there had been time for optimal management to already be found. As pointed out earlier in the paper, part of the information provided to the APR depended on the accuracy of the memory capacity of the WWE involved.

## 5 CONCLUSION

Giving birth to a malformed baby following intrauterine ASM exposure substantially increases the risk of giving birth to another malformed one in the immediately following ASM‐treated pregnancy. The risk appears several times greater if the mother has a genetic generalized epilepsy rather than a focal one. Whether changing the ASMs apparently involved would reduce that risk is unknown, but a change in ASM to a potentially less teratogenic one seems a reasonable precautionary move in that situation.

## AUTHOR CONTRIBUTIONS

**Frank Vajda:** Writing–review and editing, supervision, project administration, investigation, conceptualization. **Terence O'Brien:** Supervision, conceptualization. **Janet Graham:** Resources, project administration, data curation. **Alison Hitchcock:** Resources, project administration, data curation. **Piero Perucca:** Writing–review and editing, supervision, investigation. **Cecilie Lander:** Supervision, project administration. **Mervyn Eadie:** Writing–original draft, formal analysis, data curation.

## FUNDING INFORMATION

The funding sources that have maintained the APR over the years are mentioned above. The data analysis and costs of writing this paper involved no direct financial support.

## CONFLICTS OF INTEREST STATEMENT

F.J.E. Vajda has received research support for the Australian Pregnancy Register from the Epilepsy Society of Australia, NHMRC, RMH Neuroscience Foundation, Epilepsy Action Australia, Sanofi‐Aventis, Eisai, UCB Pharma, Jazz Pharmaceuticals, and Sci‐Gen. T. O'Brien has received research support from the Epilepsy Society of Australia, NHMRC, RMH Neuroscience Foundation, Sanofi‐Aventis, UCB Pharma, and Sci‐Gen, and Eisai. P. Perucca is supported by an Emerging Leadership Investigator Grant from the Australian National Health and Medical Research Council (APP2017651), the University of Melbourne, Monash University, the Weary Dunlop Medical Research Foundation, Brain Australia, and the Norman Beischer Medical Research Foundation. He has received speaker honoraria or consultancy fees to his institution from Chiesi, Eisai, LivaNova, Novartis, Sun Pharma, Supernus, and UCB Pharma, outside the submitted work. He is an Associate Editor for Epilepsia Open. J.E. Graham, A.A. Hitchcock, C.M. Lander, and M.J. Eadie have no relevant conflicts of interest to declare.

## CONSENT

Informed consent for participation was provided by women at the time of their inclusion in the APR's database.

**Test Yourself**

1. Based on the information contained in the paper on Consecutive Pregnancies in women with epilepsy: Which one or more of the following are incorrect:
  - When there had been no change in ASM therapy between consecutive pregnancies, freedom from seizures was more likely in the second pregnancy than in the first for women with epilepsy of all types
  - When there had been no change in ASM therapy between consecutive pregnancies, freedom from seizures was more likely in the second pregnancy than in the first, but only for women with generalized epilepsies
  - When there had been no change in ASM therapy between consecutive pregnancies, freedom from seizures was more likely in the second pregnancy than in the first, but only for women with focal epilepsies
  - When there has been a change in ASM therapy between consecutive pregnancies, freedom from seizures is less likely in the second pregnancy than in the first, but only for women with generalized epilepsies
  - When there had been a change in ASM therapy between consecutive pregnancies, freedom from seizures was less likely in the second pregnancy than in the first, but only for women with focal epilepsies
2. Based on the information contained in the paper on Consecutive Pregnancies in women with epilepsy: In consecutive pregnancies where fetal malformation occurred in at least one member of the pair, which one or more of the following ASMs had a substantially higher rate of use in the second of each pair of pregnancies?
  - Carbamazepine
  - Lamotrigine
  - Levetiracetam
  - Topiramate
  - Valproate
3. Based on the information contained in the paper on Consecutive Pregnancies in women with epilepsy: When a malformed fetus had resulted from the first of a pair of consecutive pregnancies, which one or more of the following were associated with an increased hazard of a malformed fetus occurring in the woman's following pregnancy?
  - Having focal epilepsy
  - Having generalized epilepsy
  - Having a seizure‐free pregnancy
  - Taking carbamazepine during pregnancy
  - Having a family history of fetal malformation

*Answers may be found in the* *supporting information*.

## Supporting information


Appendix S1.

## Data Availability

The data on which this paper is based are held in an unidentified form in the APR database currently housed in the Royal Melbourne Hospital, Melbourne, Australia. It should be noted that the data have been provided in stages to the EURAP database, and that parts of it (which we cannot identify) may have already been used in publications emanating from EURAP.
