# Supplementary material for: The outcomes of consecutive pregnancies in Australian women with epilepsy
Source: Epileptic Disord. 2026 May 15;28(4):1211–20. doi: 10.1002/epd2.70265 (PMC13499245; doi:10.1002/epd2.70265)
Supplement: Supplementary file 1 [file EPD2-28-1211-s001.docx]

Answer Key

Question 1	Answer: D, E

Question 2	Answer: C

Question 3	Answer: B, E
